# Diabetic striatopathy: A case report of haemichorea in an older adult woman with poorly controlled diabetes

**DOI:** 10.51866/cr.1013

**Published:** 2026-03-08

**Authors:** Nadiah Abdul Rahim, Say Yee Loo, Saharuddin Ahmad, Ezura Madiana Md Monoto, Hana Azhari

**Affiliations:** 1 Klinik Primer HCTM, UKM, Department of Family Medicine, Jalan Dwitasik, Bandar Sri Permaisuri, Cheras, Kuala Lumpur, Malaysia.; 2 Department of Family Medicine, Faculty of Medicine, 14th Floor, Pre-clinical Block, Universiti Kebangsaan Malaysia, Jalan Yaacob Latif, Bandar Tun Razak, Cheras, Kuala Lumpur, Malaysia.

**Keywords:** Chorea, Diabetic mellitus, Type 2

## Abstract

Uncontrolled type 2 diabetes (T2D) is associated with numerous microvascular and macrovascular complications. While clinical focus has often been on acute emergencies such as diabetic ketoacidosis and hyperosmolar hyperglycaemic state, rarer neurological complications also warrant attention. One such under-recognised complication is diabetic striatopathy (DS), also referred to as chorea–hyperglycaemia–basal ganglia syndrome or hyperglycaemia-induced chorea. We present the case of an older adult woman with poorly controlled T2D who developed progressive right-sided involuntary limb movements with twitching of the mouth. Clinical assessments and investigations confirmed DS, and her symptoms resolved completely with glycaemic optimisation and short-term risperidone for symptom relief. This case highlights the importance of recognising this uncommon presentation of uncontrolled T2D in the primary care setting, where timely diagnosis, as well as effective glycaemic optimisation, can lead to full recovery and a good prognosis.

## Introduction

Diabetic striatopathy (DS) is characterised by hyperglycaemia in association with one or both of the following: (1) acute-onset chorea–ballism and (2) striatal hyperdensity on CT or striatal hyperintensity on Tl-weighted MRI.^1-3^ This definition is based on patterns consistently reported in the literature, as no major neurological or endocrine society has yet established formal diagnostic criteria for DS.

The condition predominantly affects older women with poorly controlled diabetes.^1,4^ The estimated global prevalence of 1 in 100,000 is likely underestimated due to limited recognition and frequent misdiagnosis as other neurological emergencies.^5^

DS was first described in 1960, and its pathophysiology remains incompletely understood.^6^ Two main theories have been proposed. The metabolic theory attributes abnormal movements to depletion of y-aminobutyric acid and acetylcholine in the basal ganglia during non-ketotic hyperglycaemia, although it does not fully explain unilateral or persistent symptoms after glucose correction or occurrence during hypoglycaemia and ketotic hyperglycaemia. The vascular theory proposes that striatal hypoperfusion related to diabetic vasculopathy or hyperviscosity, leads to choreiform movements. Both theories highlight the combined metabolic and vascular involvement of the basal ganglia.^4^

Most reported cases originated from Asia (71.6%), with fewer cases documented in Europe and the Americas. Within Southeast Asia, Malaysia contributed only 0.42% of reported cases, reflecting the rarity and under-recognition locally.^1^ Furthermore, the predominance of reported cases from tertiary hospital settings, where advanced neuroimaging and specialist input are more readily available^7,8^, suggests that the true disease burden is underestimated, particularly at the primary care level. We report a case identified in a primary care clinic during the assessment of a patient with worsening glycaemic control.

## Case presentation

An 87-year-old woman with long-standing type 2 diabetes (T2D), hypertension, dyslipidaemia and chronic kidney disease stage 4 presented with continuous, irregular and non-patterned movements of her right upper limbs accompanied with random jerks of her lower limbs and twitching at the right corner of her mouth for 3 weeks. Her condition was associated with worsening abnormal movements involving the right arm, leg and face which were present throughout the day and disappeared during sleep. She denied fever, constitutional symptoms, osmotic symptoms, acute behavioural changes or cognitive decline. She had no personal or family history of movement disorders, and there had been no change in her regular medications, none of which were known to cause extra-pyramidal side effects. There was no preceding trauma, and she had otherwise been well except for worsening home blood glucose levels, which ranged around 14–16 mmol/L. Her blood pressure upon presentation was 144/69 mmHg, while her random capillary sugar level was 14.2 mmol/L. Electrocardiogram demonstrated sinus rhythm at a rate of 72 bpm with premature atrial complexes.

Neurological examination confirmed right sided choreiform movements with dystonia and orofacial dyskinesia. Muscle tone was preserved, with normal power, reflexes, coordination, cranial nerve function and mental status.

Her blood parameters were summarised in Table 1.

**Table 1:** Summary of the patient’s blood parameters upon presentation.

Test	Result	Unit
Random blood sugar	14.2	mmol/L
HbAlc	10.3	%
HCO3	22.7	mmol/L
Lactate	1.9	mmol/L
Urine ketone	Neg	mmol/L
Hemoglobin	10.0	g/dL
Platelet	220	x10^9/L
Total white cell	8.2	x10^9/L
Urea	12.8	mmol/L
Sodium	138	mmol/L
Potassium	5.1	mmol/L
Creatinine	184	mmol/L
Calcium	2.39	mmol/L
Magnesium	0.75	mmol/L
Phosphate	1.33	mmol/L

**Figure 1:**
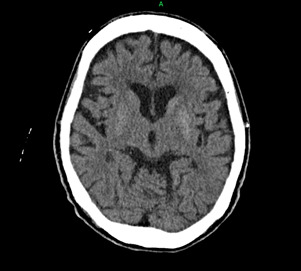
Non-contrast brain CT scan of the patient showing subtle hyperdensity of the left striatum without evidence of mass effect, oedema or volume loss and with no sign of brain ischaemia.

She was referred to the emergency department for brain CT to exclude intracranial pathology. The non-contrast CT scan demonstrated subtle hyperdensity in the left striatum (Figure 1), a classical though not consistently present finding in DS. This pattern helped exclude ischaemic or haemorrhagic stroke.

A diagnosis of DS was made. The patient was co-managed with the neuromedical team. She was prescribed oral hypoglycaemic agents (glimepiride and linagliptin) and a tapering dose of risperidone. Her abnormal movements improved within 1 week and resolved completely after 3 weeks. The patient’s glycaemic control was closely monitored in primary care, with home blood glucose levels ranging from 10 to 12 mmol/L. Three months later, her HbAlc level improved to 8.4%.

## Discussion

DS is a rare but increasingly recognised neurological complication of poorly controlled diabetes, characterised by hyperkinetic movement including haemichorea and haemiballism.^9,10^ Haemichorea is described as brief, continuous, non-rhythmic, irregular, involuntary movements and haemiballism as proximal, larger, flinging movements of the limbs. Both conditions typically affect one side of the body.^11^ DS predominantly affects older, Asian, and female patients, a profile that closely matches our case. This condition can occur regardless of diabetes type as it is related to the presence of severe non-ketotic hyperglycaemia.^12^ With Malaysia’s ageing population and rising prevalence of T2D, the occurrence is likely to increase, requiring greater clinical awareness, particularly among primary care physicians.

The differential diagnosis of chorea is challenging in primary care and maybe broadly classified into hereditary and non-hereditary causes, with further subdivision into acute (stroke or intracerebral haemorrhage), subacute (metabolic disorders, drug-induced causes or malignancies) and chronic (neurodegenerative diseases). Asymmetric presentations often suggest a structural or metabolic cause.^11^ In our patient, the acute onset of unilateral choreiform movements with markedly elevated blood glucose levels, pointed towards a metabolic aetiology consistent with DS.

A structured diagnostic approach is essential, beginning with detailed history-taking to establish onset, progression and potential triggers should be followed by a targeted neurological examination to distinguish chorea from other hyperkinetic disorders such as dystonia or myoclonus.^13^ In older adults with diabetes, new-onset involuntary movements should raise the suspicion for metabolic causes besides more commonly acquired causes, including acute stroke, infection and drug-induced causes. This can be supported by medication review, laboratory work-up and basic neuroimaging studies. Arecco et al. emphasised the importance of assessing both blood glucose and HbA1c levels in such presentations.^4^

In Flow Charts 1 and 2, we propose a practical algorithm to guide primary care clinicians in evaluating adults who present with abnormal movements.

**Flow chart 1:**
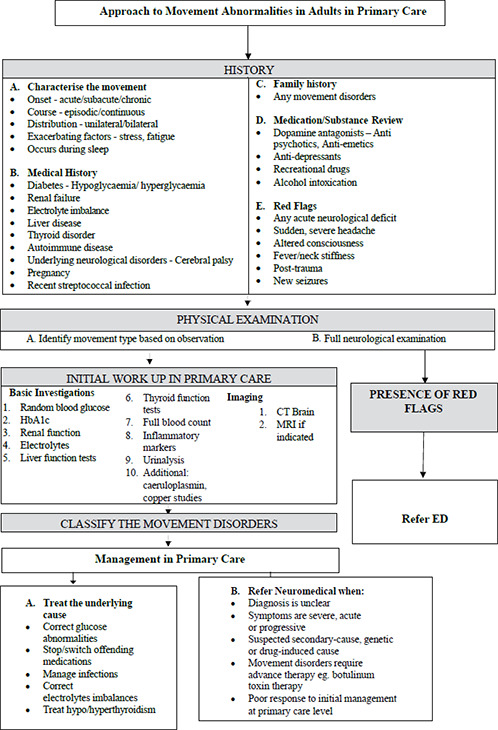
Approach to movement abnormality in primary care^14-16^

**Flow chart 2:**
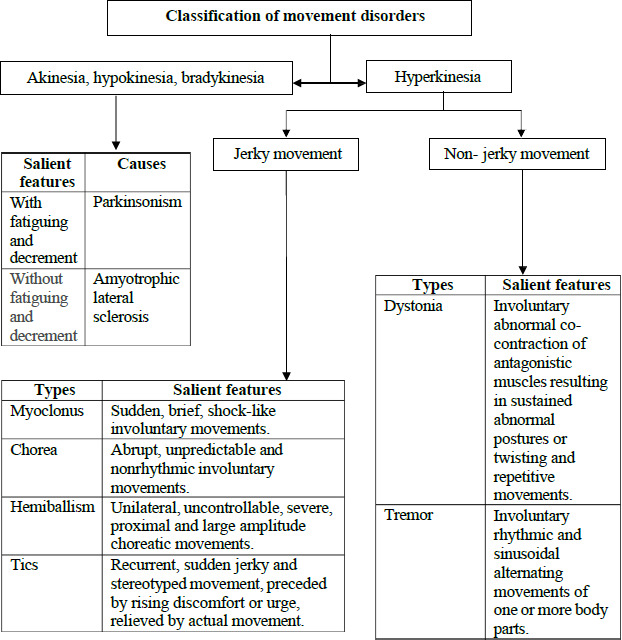
Classification of movement disorders^16^

The typical neuroradiological features of DS include striatal hyperdensity on CT and hyperintensity on T1-weighted MRI.^17,18^ In our patient, the non-contrast brain CT demonstrated subtle hyperdensity in the striatum (Figure 1). In a series of 59 patients with acute dyskinesia, Dubey et al. reported abnormal striatal signals in only 44.1% while 55.9% had normal neuroimaging findings, highlighting that imaging findings are often absent in DS.^2^

CT imaging also helps to exclude ischaemic or haemorrhagic stroke. DS characteristically shows unilateral striatal hyperdensity without mass effect, oedema or midline shift, reflecting metabolic dysfunction rather than haemorrhage.^1,19^ In comparison, ischaemic stroke typically manifests as hypodensity within a defined vascular territory, with loss of grey-white matter differentiation or sulcal effacement. Haemorrhagic stroke presents as a well-demarcated hyperdense collection with surrounding oedema, mass effect or intraventricular extension, features that are not seen in DS.^17^ Thus, the CT findings are keys to differentiating DS from stroke, particularly in older adult patients with diabetes presenting with acute movement disorders.

Although MRI is more sensitive in detecting the characteristic T1 hyperintensity of DS,^1^ it was not performed in this case because urgent intracranial pathology had been excluded by CT and the diagnosis was supported by clinical and biochemical evidence. This pragmatic approach is often necessary in resource-conscious primary care settings.

Management of DS focuses on glycaemic stabilisation and controlling choreiform movements. Glycaemic optimisation alone leads to clinical improvement in approximately 25.7% of cases, while the addition of antichorea medications increases the overall success rate to 76.2%.^1^ Clinical evidence supports the use of haloperidol and risperidone for symptomatic relief, as their striatal D2-receptor antagonism temporarily modulates the dopaminergic imbalance which was thought to drive chorea, hence reducing the severity and frequency of chorea.^17^ In our patient, her symptoms were disabling and interfered with daily activities; hence, a short course of risperidone was initiated to provide temporary symptomatic relief during recovery.

The overall prognosis of DS is favourable, with most patients achieving full recovery.^1,4,11^ However, the recurrence rate is reported as 8.2%, highlighting the need for long-term diabetic control.^1^ In our patient, complete resolution was observed on follow-up, highlighting the reversible nature of this condition when promptly identified and treated.

This case highlights several key learning points for primary care physicians. First, chorea in older adult patients should trigger a focused evaluation of metabolic causes, particularly uncontrolled diabetes. Second, basic investigations available at primary care facililties can establish the diagnosis and guide management without immediate recourse to advanced imaging. Third, timely glycaemic control, with short-term symptomatic therapy when required, can lead to full recovery. Finally, raising awareness of DS among frontline clinicians may facilitate earlier recognition and improved patient outcomes in Malaysia’s ageing population with diabetes.

## Conclusion

DS is a rare yet reversible cause of acute chorea in older adult patients with diabetes. This case highlights the importance of recognising DS as a reversible cause of acute chorea in older adult patients with diabetes. Primary care physicians are well positioned as frontliners in the early recognition, confirmation and management of this rare condition, which can result in an excellent prognosis.

## References

[1] CB Chua, CK Sun, CW Hsu, YC Tai, CY Liang, IT Tsai (2020). Diabetic striatopathy: clinical presentations, controversy, pathogenesis, treatments, and outcomes. Sci Rep.

[2] S Dubey, S Chatterjee, R Ghosh (2022). Acute onset movement disorders in diabetes mellitus: A clinical series of 59 patients. Eur J Neurol.

[3] Y Abe, T Yamamoto, T Soeda (2009). Diabetic striatal disease: clinical presentation, neuroimaging, and pathology. Intern Med.

[4] A Arecco, S Ottaviani, M Boschetti, P Renzetti, L Marinelli (2024). Diabetic striatopathy: an updated overview of current knowledge and future perspectives. J Endocrinol Invest.

[5] WG Ondo (2011). Hyperglycemic nonketotic states and other metabolic imbalances. Handb Clin Neurol.

[6] SF Bedwell (1960). Some observations on hemiballismus. Neurology.

[7] B Johari, M Hanafiah, AM Shahizon, M Koshy (2014). Unilateral striatal CT and MRI changes secondary to non-ketotic hyperglycaemia. BMJ Case Rep.

[8] SL Fong, AH Tan, KF Lau, N Ramli, SY Lim (2019). Hyperglycemia-Associated Hemichorea-Hemiballismus with Predominant Ipsilateral Putaminal Abnormality on Neuroimaging. J MovDisord.

[9] ML Cheneler, K Qureshi, C Bahrami (2024). A case of diabetic striatopathy due to uncontrolled type 2 diabetes. Endocrinol Diabetes Metab Case Rep.

[10] F Nilofar, G Ganapathy, S Bose, V V (2024). Unraveling Diabetic Striatopathy: Clinical and Imaging Perspectives. Cureus.

[11] C Saft, JM Burgunder, M Dose (2023). Differential diagnosis of chorea (guidelines of the German Neurological Society). Neurol Res Pract.

[12] A Yasuhara, J Wada, H Makino (2008). Bilateral dystonia in type 1 diabetes: a case report. J Med Case Rep.

[13] E Furr Stimming, Z Zeier, J Patino (2024). Clinical approach to the diagnostic evaluation of chorea. Pract Neurol.

[14] JM Longmore, E Al (2010). Abnormal involuntary movements. Oxford Handbook of Clinical Medicine.

[15] M Bouchard, O Suchowersky (2024). Overview of chorea. UpToDate.

[16] WF Abdo, BP van de Warrenburg, DJ Burn, NP Quinn, BR Bloem (2010). The clinical approach to movement disorders. Nat Rev Neurol.

[17] SH Oh, KY Lee, JH Im, MS Lee (2002). Chorea associated with non-ketotic hyperglycemia and hyperintensity basal ganglia lesion on T1-weighted brain MRI study: a meta-analysis of 53 cases including four present cases. J Neurol Sci.

[18] M Dong, JY E, L Zhang, L Teng W Tian (2021). Non-ketotic hyperglycemia choreaballismus and intracerebral hemorrhage: a case report and literature review. Front Neurosci.

[19] C Cosentino, L Torres, Y Nunez, R Suarez, M Velez, M Flores (2016). Hemichorea/hemiballism associated with hyperglycemia: report of 20 cases. Tremor Other HyperkinetMov (NY).

